# A role for *MIR828* in pineapple fruit development

**DOI:** 10.12688/f1000research.21779.2

**Published:** 2020-04-08

**Authors:** Christopher D. Rock

**Affiliations:** 1 Department of Biological Sciences, Texas Tech University, Lubbock, TX, 79409, USA

**Keywords:** anthocyanins, microRNAs, fruit development, RNA interference, evolution

## Abstract

Chen *et al. *( *Nature Genet*. 51: 1549–1558; Oct. 2019) sequenced *Ananas comosus* var. *bracteatus* accession CB5, cultivated for its bright pink-to-red colored fruit, and yellow-fleshed *A. comosus* accession F153, reporting an improved F153 reference assembly while annotating *MICRORNA (MIRNA)* loci and gene family expressions relevant to lignin and anthocyanin biosynthesis. An independent article (Xiong *et al.* *Sci. Rep*. 8: 1947; 2018) reported var. *bracteatus MIRNAs *but not *MIR828*, a negative regulator of anthocyanin and polyphenolics biosynthesis by targeting *MYB* transcription factors associated with UV light- and sugar-signaling in dicots. *MIR828* has been reported in gymnosperms, Amborella (sister to flowering plants), and basal monocot orders Liliales, Asparagales, Zingiberales, Arecales, but not in the Poales, a sister order comprising grasses and ~3,000 species of bromeliads including pineapple. Here I show *MIR828* exists in pineapple and directs post-transcriptional gene silencing of mRNAs encoding MYB family members with inferred function to regulate the conspicuous red fruit trait in var. *bracteatus*. *MIR828* plesiomorphy (an ancient basal trait) may shed light on monocot apomorphic fruit development, postulated for 21 monocot families with fleshy fruits as due to homoplasy/convergence driven by tropical climate and/or enticements to vertebrate endozoic seed dispersers.

Chen *et al*. ^1^ sequenced *Ananas comosus* var *. bracteatus* accession CB5, cultivated for its bright pink-to-red colored fruit, and yellow-fleshed *A. comosus* accession F153, reporting an improved F153 reference assembly ^2^ while annotating *MICRORNA* ( *MIRNA*) loci ^2– 4^ and gene family expressions relevant to lignin and anthocyanin biosynthesis. An independent article reported var. *bracteatus MIRNA*s ^5^ but not *MIR828* ^6^, a negative regulator of anthocyanin and polyphenolics biosynthesis by targeting *MYB* transcription factors associated with UV light- and sugar-signaling in dicots ^7, 8^. *MIR828* has been reported in gymnosperms ^8^, Amborella (sister to flowering plants) ^9^, and basal monocot orders Liliales ^8, 10^, Asparagales ^11^, Zingiberales ^12, 13^, Arecales ^14^, but not in the Poales, a sister order comprising grasses and ~3,000 species of bromeliads including pineapple ^15^. Here I show *MIR828* exists in pineapple and directs post-transcriptional gene silencing of mRNAs encoding MYB family members with inferred function to regulate the conspicuous red fruit trait in var. *bracteatus*. *MIR828* plesiomorphy (an ancient basal trait) may shed light on monocot apomorphic fruit development, postulated for 21 monocot families with fleshy fruits as due to homoplasy/convergence driven by tropical climate and/or enticements to vertebrate endozoic seed dispersers ^16^.

The astronomer Carl Sagan popularized the quip (a corollary of Occam’s Razor) “ *absence of evidence is NOT evidence of absence*.” Taking a conservative approach applies especially to quantitative transcriptomics. For example, the existence of regulatory and post-transcriptional processes increase the complexity of non-coding RNA space, where annotation is sparse. Analysis of Chen *et al*’s *A. comosus* MD-2 cultivar leaf small RNA (sRNA) libraries and stranded RNA-seq libraries ^4^ from flowers and fruits of F153 and CB5 genotypes establish the existence of aco-miR828 ( *Extended data:* Figure S1 ^17^) and *pri-MIR828* expressions, with the novel observation that *pri-MIR828* is properly transcribed in leaves, flowers and fruits yet ~50% of mature miR828 species abundance (0.3 reads per million in leaves from 264.7 million reads; *Extended data:* Table S1 ^17^) are 21 nt, while 93% of the equally abundant 22 nt species appear to have undergone non-templated 3’ uridylation of the 21 nt species ^18^ ( *Extended data:* Table S1 ^17^). Analysis of independent bracteatus cultivar leaf sRNA libraries (NCBI SRA SRR5677552-7; 113.2 million reads) ^5^ of unknown provenance relative to the subject CB5 genotype failed to identify any *MIR828* reads.

Fortuitously, a unique aspect of miR828 is that despite its very low abundance, it has very high activity ^7, 19^ that serves as diagnostic. miR828 guides ARGONAUTE slicing of target *MYB* mRNAs within the deeply conserved SANT domain region ^8^ by Watson-Crick complementarity, with consequent knock-on production of easily quantified DICER-mediated sense- and antisense 21 nt phased small-interfering RNAs (phasiRNAs) mapping downstream (3’) on target *MYB* transcripts. The improved F153 reference assembly ^1^ contains two candidate miR828-targeted *MYB*s: Aco017254.1 (LG4), with two introns of 1113 and 1340 nts, and Aco020986.1 (LG14) without RNA-seq evidence of intron splicing, whereas the CB5 bracteatus genome only contains one Aco017254.1 homologous gene with RNA-seq splicing evidence for conserved introns of 1109 and 1332 nt ( *Extended data:* Table S2 ^17^). When phasiRNA expressions from leaf sRNA libraries ^4, 5^ are respectively mapped to the F153 and CB5 candidate *MYB* target mRNAs, it is apparent that F153 *MYB* transcripts clearly undergo miR828-guided slicing, evidenced by D1(+) phased siRNA reads mapping to the 10 ^th^ nucleotide position of miR828 homology to the mRNA target, and unique sense and antisense secondary phasi-RNAs mapping precisely in multiples of 21 nt downstream from the detected slice sites ( Figure 1; *Extended data:* Figure S2, Table S3 ^17^). The CB5 reference genome target *MYB* locus Aco017254.1 homolog (contig tig00012294, CABWKS010000088.1:25590-28868rc) encodes two missense codons at residues 41 (M➔R) and 200 (N➔K compared to F153), four silent codon substitutions, and RNA-seq analysis reveals mis-annotation of the CB5 genome which lacks two Gs (at contig residues 28780 and 27566) that exist and result in a CB5 open reading frame of the same size as Aco017254.1 in F153, including six instead of seven trinucleotide GGC glycine codon repeats templated in the CB5 genome at residue 204 ( *Extended data:* Table S2 ^17^).

**Figure 1.**
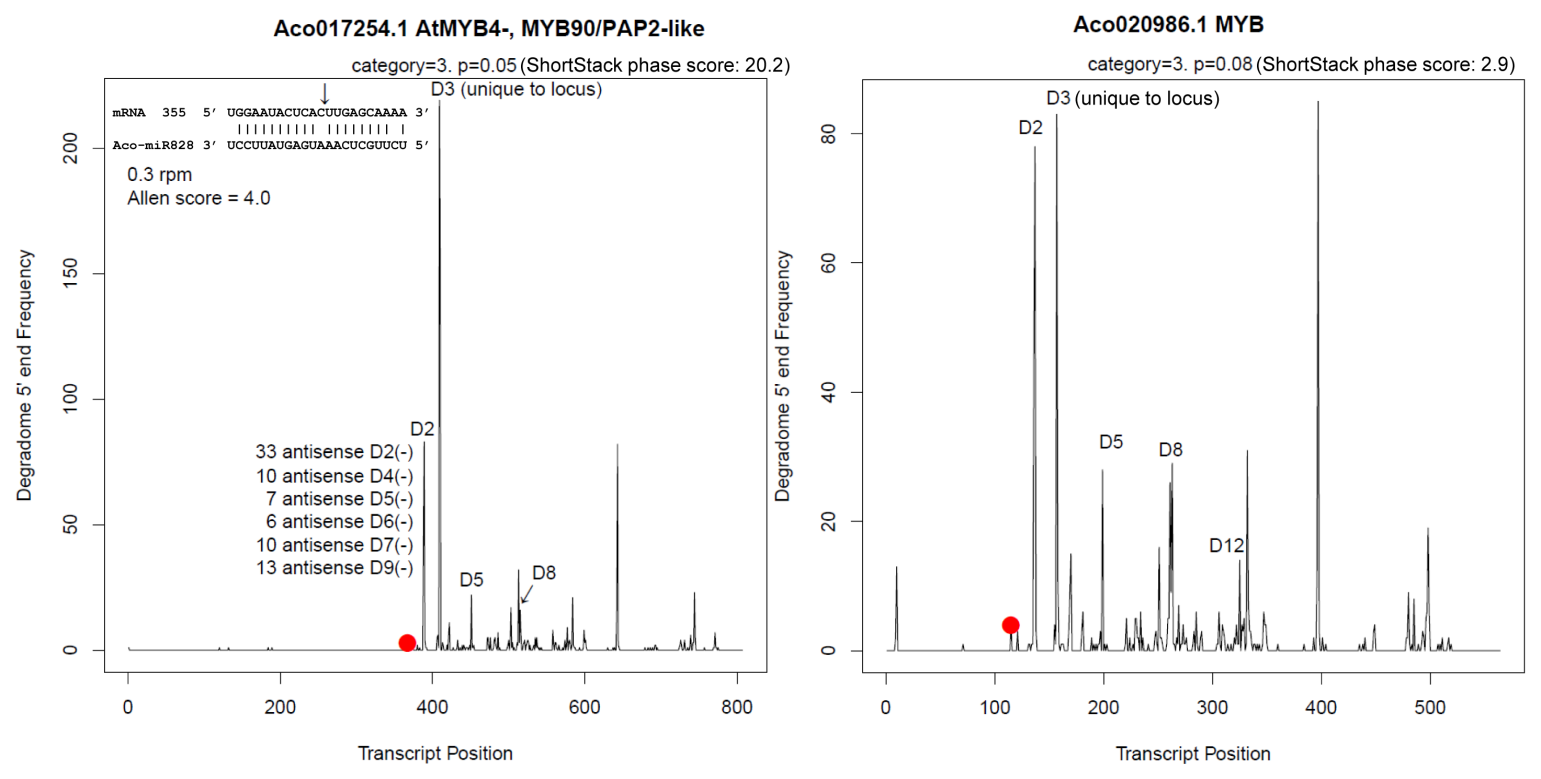
Pseudo-degradome (from sRNA libraries) T plots of 5’ ends of sRNA alignments show post-transcriptional gene silencing of miR828 *MYB* targets in MD-2 leaf sRNA libraries ^4^. Concatenated sRNA libraries were used as pseudo-degradome on grounds sliced mRNAs subject to production of amplified diced dsRNAs are manifest in sRNA libraries ^7, 20, 21^. Slicing is at nt10 from 5' end of miR828 (arrow, inset; same target sequence for Aco020986.1 at mRNA nt 115). Y axis is numbers of reads in sRNA libraries mapped to cDNAs; red dot is the documented miR828 sliced sRNA species that sets the register for 3’ phasiRNA production.

In contrast to demonstrated post-transcriptional silencing activity of miR828 on target *MYB* abundance in F153 leaf samples ( Figure 1), analysis of six bracteatus leaf sRNA libraries from an independent study ^5^ did not provide any evidence of target *MYB* Aco017254.1 mRNA slicing or target phasiRNA accumulation ( *Extended data:* Table S3 ^17^). Taken together, subject to the caveat that the provenance of the bracteatus cultivar used for the sRNA analysis ^5^ may be different than subject CB5 genotype, the data suggest there may be differences in expression and/or regulation of *pri-MIR828*, and/or target *MYB* Aco017254.1 between F153 yellow-fleshed versus CB5 red-fleshed genotypes. Figure 2 shows this is indeed the case in various tissues examined, with the evidence supporting higher *pri-MIR828* expression in F153 ovules concordant with lower target *MYB* Aco017254.1 mRNA levels ( Figure 2A), significant decreases over time from stage 1 early fruit development for target *MYBs* Aco017254.1 and Aco020986.1 in yellow-fleshed MD-2 cultivar ( Figure 2B), whereas in contrast there is a trend of lower expression of CB5 *pri-MIR828* concordant with sustained higher abundance of Aco017254.1 target *MYB* ( Figure 2C stage 7 versus stage 1) than seen in MD-2 during the ripening stage of red-fleshed CB5 genotype (compare Figure 2C stage 7 showing high CB5 Aco017254.1 abundance to Figure 2B stage 7 showing very low MD-2 Aco017254.1 abundance).

**Figure 2.**
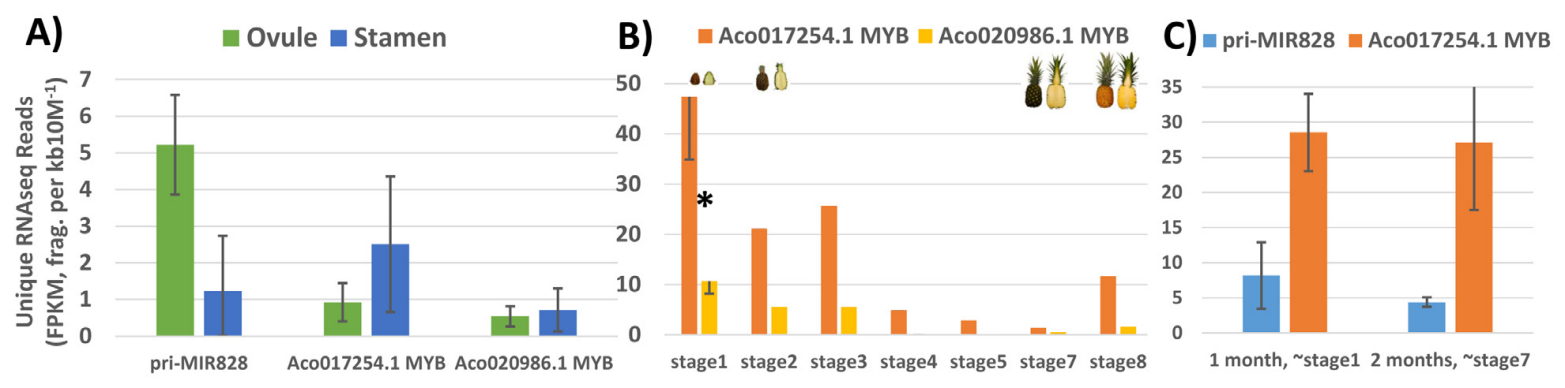
Evidence for relatively different expressions of *pri-MIR828* and target *MYB* Aco017254.1, Aco020986.1 mRNAs in yellow-fleshed F153 (A, B) versus (C) red-fleshed CB5 reproductive tissues ^1^. **A**) Inverse relationship of *pri-MIR828* (high; see *Extended data*: Table S1, rows 23–36) to target *MYB* Aco017254.1 (low *Extended data*: Table S2, rows 406–470) anthocyanin effector expressions in F153 ovules. Error bars are s.e.m., n=3 biological replicates. **B**) Down regulation of miR828-targeted *MYB* anthocyanin effectors during MD2 yellow-fleshed fruit development (n=1 per stage *Extended data*: Table S2, rows 134–405)). Asterisk (*) denotes stage 1 significantly different across Aco017254.1 and Aco020986.1 than stages 2-8, *p* = 0.02 (Student’s two-sided paired t-test, equal variance assumed). Stage 1 error bars show the 95% confidence interval for significance. **C**) Apparent maintenance of positive anthocyanin effector *MYB* Aco017254.1 expression at “ripe” two-month stage of CB5 red-fleshed fruit development, concordant with trend of lower *pri-MIR828* expression (see *Extended data*: Table S1, rows 37–52; Table S2, rows 471–883). Error bars are s.e.m., n=5 biological replicates.

Plant development gives rise to an astounding complexity of shapes, colors, and functions that Darwin called ‘an abominable mystery’ in his efforts to integrate species complexity with the unifying theory of evolution. The observations reported here potentially offer insight into the conservation of a developmental control mechanism whereby miR828 in pineapple, like in dicots silences MYBs inferred to act as positive effectors of anthocyanin biosynthesis that could give rise to the red-fleshed trait in the bracteatus variety. Consistent with this view is the finding from genome-wide functional phylogenomic approaches that ARGONAUTE1, RNA-DEPENDENT RNA POLYMERASE6, and mRNA export factor homolog SILENCING DEFECTIVE5, each required for trans-acting siRNA accumulation, played significant roles in the evolution of monocot metabolic and developmental traits ^22, 23^. Also of interest in this context is the claim of bracteatus genome authors ^24^ that convergent expansion in several Crassulacean Acid Metabolism (CAM) bromeliad lineages of *XAP5 CIRCADIAN TIMEKEEPER/XCT*, a nuclear-localized regulator of blue light responses ^25^ and sRNA production ^26^, supports evolution of the myriad metabolic and physiological transitions from C3 to CAM photosynthesis by duplication/differentiation of a highly pleiotropic effector ^24^.

## Data availability

### Underlying data

RNA-seq data ^1^, CB5 reference genome GCA_902506285.1, and F153 improved assembly genome GCA_902162155.1 were downloaded from NCBI via BioProject accession code PRJEB33121. sRNA raw data ^5^ for *A. comosus* var. *bracteatus* leaf libraries were downloaded from NCBI BioProject accession code PRJNA389361. Processed data ^4^ for MD-2 leaf sRNA libraries Pn_Gr2am_1, Pn_Gr3pm_3, Pn_Gr4pm_1, Pn_Gr4pm_3, Pn_Gr6am_2, Pn_GrMid_3, Pn_Wh10am_2, Pn_Wh10pm_1, Pn_Wh1pm_1, Pn_Wh2am_1, Pn_Wh2am_2, Pn_Wh3pm_1, Pn_Wh3pm_2, Pn_Wh4am_1, Pn_Wh4pm_1, Pn_Wh6am_1, Pn_Wh6am_2, Pn_Wh6pm_1, Pn_Wh6pm_2, Pn_Wh8am_2, Pn_Wh8pm_2, Pn_WhMid_1, and Pn_WhMid_2 were downloaded from https://mpss.danforthcenter.org/dbs/index.php?SITE=pineapple_sRNA.

The original *A. comosus* F153 reference genome and cDNA fasta file ‘Acomosus_321_v3.cds.fa’ can be browsed online and downloaded at https://phytozome.jgi.doe.gov/ ^27^.

### Extended data

Figshare: A role for MIR828 in pineapple fruit development, https://doi.org/10.6084/m9.figshare.11388051.v2 ^17^

This project contains the following extended data:

- Figure S1. strucVis graphical linear output of Aco- *MIR828* hairpin structure and sRNA abundance evidence from 23 MD-2 leaf sRNA libraries.
- Figure S2. PhaseTank alignment output of leaf MD-2 sRNA libraries mapped to candidate miR828 target MYB cDNAs, F153 improved reference genome.
- Table S1: Evidences for *pri-MIR828* abundance in F153 and CB5 varieties.
- Table S2: RNA-seq evidence for miR828 target *MYB* abundance.
- Table S3: bowtie mapping of sRNAs to miR828 target *MYB* mRNAs

Data are available under the terms of the Creative Commons Zero "No rights reserved" data waiver (CC0 1.0 Public domain dedication).

## Software availability

Fastx-toolkit version 0.0.14 for trimming Illumina universal adapterAGATCGGAAGAGCACACGTCTGAACTCCAGTCA (fastx_clipper option -l 18; discards short reads) and fasta file manipulations (fastx_uncollapser to expand the pre-processed data ^4^; fastq_to_fasta for inputs to CleaveLand) is available at http://hannonlab.cshl.edu/fastx_toolkit/. FastQC version 0.11.5 for quality control of fastq raw sequence data is available at https://www.bioinformatics.babraham.ac.uk/projects/fastqc/. ShortStack ^28^ version 3.8.5 (options --mincov 15 --foldsize 340; *Extended data*: Figure S1, Table S1 ^17^) for comprehensive annotation and quantitation of *MIRNA*s and sRNA cluster phasing is available at . strucVis for visualization of predicted RNA secondary structures with overlaid sRNA depths display of ShortStack output ( *Extended data*: Figure S1, page 2) is available at https://github.com/MikeAxtell/strucVis. CleaveLand ^29^ version 4.4 for plotting 5’ ends of sRNA libraries as pseudo-degradome inputs (amplified sense and anti-sense siRNAs in phase and derived from miRNA-sliced mRNAs ^7, 20, 21^) to find sliced miRNA targets is available at https://github.com/MikeAxtell/CleaveLand4. PhaseTank ^30^ version 1.0 for quantifying and aligning phasiRNAs to miRNA target mRNAs ( *Extended data*: Figure S2 ^17^) is available at http://phasetank.sourceforge.net/; the linux command sed -i ‘s/-/_x/’ was used to reconfigure fastx_collapser output from concatenated sRNA fasta library files to PhaseTank input style “>t1_xN” where N is number of collapsed reads). Magic-BLAST ^31^ version 1.5.0 for RNA-seq fastq read alignment ( *Extended data*: Table S2 row 4 for options parameters ^17^) to reference genomes is available at https://ncbi.github.io/magicblast/. Blastn ^32^ version 2.6.0 is available at ftp://ftp.ncbi.nlm.nih.gov/blast/executables/blast+/LATEST/. Bowtie ^33^ version 1.1.2 for short read alignment (option -v 1 to allow one mismatch) is available at http://bowtie-bio.sourceforge.net/manual.shtml. RNAfold ^34^ web server was used for generating miR828 hairpin graphics ( *Extended data*: Figure S1 ^17^) at http://rna.tbi.univie.ac.at/cgi-bin/RNAWebSuite/RNAfold.cgi. The options parameters used for various algorithms are detailed in *Extended data*: Tables S1–S3
