# Supplementary material for: A role for MIR828 in pineapple fruit development
Source: F1000Res. 2020 Apr 8;9:16. Originally published 2020 Jan 13. [Version 2] doi: 10.12688/f1000research.21779.2 (PMC7194464; doi:10.12688/f1000research.21779.2)
Supplement: Peer review history

# Peer review: Reviewer response for version 2

**DOI:** 10.5256/f1000research.25483.r62125

**Published:** 2020-04-22

**Authors:** Nathan Johnson

**Affiliations:** 1 Intercollege PhD Program in Plant Biology, Huck Institutes of the Life Sciences, Pennsylvania State University, University Park, PA, USA

I have no further comments. I feel the author has adequately addressed my concerns both in their response and in changes to text and figures.

 Thank you for your diligence and thanks for publishing this important work.

Are arguments sufficiently supported by evidence from the published literature or by new data and results?

Partly

Is the conclusion balanced and justified on the basis of the presented arguments?

Partly

Is the rationale for commenting on the previous publication clearly described?

Yes

Are any opinions stated well-argued, clear and cogent?

Yes

Reviewer Expertise:

Small RNA analysis and annotation in plants.

I confirm that I have read this submission and believe that I have an appropriate level of expertise to confirm that it is of an acceptable scientific standard.

# Peer review: Reviewer response for version 1

**DOI:** 10.5256/f1000research.24007.r60755

**Published:** 2020-03-24

**Authors:** Nathan Johnson

**Affiliations:** 1 Intercollege PhD Program in Plant Biology, Huck Institutes of the Life Sciences, Pennsylvania State University, University Park, PA, USA

This article focuses on identifying the presence of a miRNA loci (MIR828) within several cultivars of pineapple. The author leverages prior sequencing data from two studies to annotate a very lowly expressed locus which is apparently MIR828, marking the first example of this miRNA family in Poales. Despite the low expression, the author supports the presence of the miRNA through identification of targeted mRNAs, shown through detection of secondary siRNAs resulting from cleavage by a 22-nt miRNA. They also provide some evidence to speculate about the role miR828 may play in pineapple coloration relative to development.

 My background is in small RNA annotation and interpretation of function in plants. I believe that this article does a good job identifying a miRNA that is challenging to find in pineapple. I also think that the author does a compelling job proving its actual function through secondary siRNAs. False negatives are always present in the computational annotation of genomes and this type of careful analysis is often needed to find exceptions that are not caught by programs using default settings.

 I believe the conclusions of this article are true and the results to be adequate, pending some revision. My criticisms of this article mainly have to do with clarity. I think that several changes must be made to make the article more transparent in the analyses performed, the language used to describe it, and the presentation of data. I do not suggest any additional experiments to support their result, but I would like a response and modification to the manuscript based on the following notes:

1. ***“Analysis of Chen et al’s A. comosus MD-2 cultivar leaf small RNA (sRNA) libraries and stranded RNA-seq libraries from flowers and fruits of F153 and CB5 genotypes establish the existence of aco-miR828 (Extended data: Figure S1) and pri-MIR828 expressions, with the novel observation that pri-MIR828 is properly transcribed in leaves, flowers and fruits yet ~50% of mature miR828 species abundance (0.3 reads per million in leaves; Extended data: Table S1) are 21 nt, while 93% of the equally abundant 22 nt species appear to have undergone non-templated 3’ uridylation of the 21 nt species (Extended data: Table S1).”***  --- This sentence does not clearly describe what “analysis” has taken place. I need to see the author describe what evidence supports the 3 conclusions listed here, both in writing and with a description of the analysis pipeline in the methods section (see below).
2. It is preferred to include a genome-browser shot showing the read coverage for the pri-miRNA regions listed in supp figure 1, as it can be difficult to identify from a table what the locus actually looks like for the reader. It also appears that many of the rna-seq reads originate from the ‘minus’ strand. This is surprising, as the hairpin is never double stranded. Could you explain this discrepancy?
3. I like that the ShortStack output is included in supp. table 1. However, it is not clear to me what each locus refers to. What is the reference genome for each of these? It appears that cluster_1381 and cluster_1104 are using the Chen *et al.* sequencing data on the older reference genome. Why have these been shown and can you explain the lack of this locus in the older genome? I’m not sure what these loci are referring to compared to the first locus shown (cluster_4352).
4. Your RPMs for this miRNA locus are extremely low (0.3 RPM), more than 10 fold lower than ShortStacks default locus identification threshold (5 RPM). This is a real lack and makes it challenging to identify this as a miRNA. Lacking a mir-star is also a problem, but might not be surprising for a low-expression locus. As this is a published miRNA you are identifying, I am inclined to agree that it is real. However, it is inappropriate to include an artificial sequence to make ShortStack call it as such, as is done in Supp. Figure 1. Please correct this and clearly identify that this locus lacks mir-star support, though is still likely a miRNA.
5.  Consider looking into the package strucVis, an add-on for RNAfold which shows nucleotide-resolution read depth on the folded diagram. I think this would greatly clarify this figure.
6. ***“Analysis of independent bracteatus cultivar leaf sRNA libraries (NCBI SRA SRR5677552-7; 113.2 million reads) of unknown provenance relative to the subject CB5 genotype failed to identify any MIR828 reads.”***  --- This is surprising! Did you also try analysis using the F153 reference, considering the provenance of this variety is unknown? I think this is important considering the considerable depth of these libraries, where we might expect around 30 reads coming from this locus. Despite the lack of a locus identified, it might be useful to look directly at the alignment through a browser, to see if any small RNAs are coming from this locus.
7. ***“...mature miR828 species abundance (0.3 reads per million in leaves; Extended data: Table S1)...”*** --- Please show the approximate read depth here also, as was shown with the Xiong et al libraries.
8. Figure 1 is labeled as a degradome, when it is actually a plot of 5’ ends of sRNA alignments, thought to be secondary siRNAs. While I agree that secondary siRNAs can be strong evidence of targeting. However, this is entirely distinct from a degradome, which are derived from isolated mRNA degradation products. Please remove references of this as a degradome and correct the language to clearly describe this. This also should contain a citation for one of the following papers: where this evidence was used for confirming targeting (Shahid *et al.* 2018 ^1^), or evidence of secondary sRNAs as a product of miRNA targeting (Allen *et al.* 2005 ^2^).
9. Figure 2 has several critical problems. In general, I think the author must provide more detail in the conclusions and evidence shown in each section of the figure. I have detailed some more specific points as follows:
  1. It is deceiving to use bar plots to represent such small numbers of data points. Bars that represent an N of ideally 5 or less are much more informative shown as points representing the actual data. To show error bars (I assume... it is not indicated if these are standard error or deviation) greatly misrepresents the variance of these data. I would like to see these figures redone showing points rather than bars, or at least showing the raw data in a supplementary table (see below).
  2. Figure 2B is vague in its description of the test, and replicates involved. The legend describes an N=1 for all stages. If this is true, how can a statistical comparison be made? Stage 1 shows error bars, does this represent N>1? Additionally, precisely which samples are being grouped and compared here for the test? This must be made clearer.
  3. The interpretation of results in Figure 2C is unclear. There is little to no textual description of what the author concludes from Figure 2 in general and it is mainly confined to one sentence where all three sub-figures are mentioned. This needs to be greatly expanded upon for me to understand what they perceive as the outcome of these analyses.
  4. I recommend including *all* of the raw data shown in these charts in supplementary tables in addition to a more clear demonstration of the data-points mentioned above. This allows the reader to clearly identify what is being shown in each.
10. A description of methods is lacking. Though the sequencing data is derived from prior publications, the analysis performed in this paper is new. You need to describe the logical steps taken for each analysis, including processing, tools used in a step, the actual commands used, and data that is output. This would go a long way to clarifying the actual analyses they performed. This needs to be shown for RNA-seq analyses, sRNA-seq analyses, and identification of pri-miRNAs.

Are arguments sufficiently supported by evidence from the published literature or by new data and results?

Partly

Is the conclusion balanced and justified on the basis of the presented arguments?

Partly

Is the rationale for commenting on the previous publication clearly described?

Yes

Are any opinions stated well-argued, clear and cogent?

Yes

Reviewer Expertise:

Small RNA analysis and annotation in plants.

I confirm that I have read this submission and believe that I have an appropriate level of expertise to confirm that it is of an acceptable scientific standard, however I have significant reservations, as outlined above.

## References

[1] : MicroRNAs from the parasitic plant Cuscuta campestris target host messenger RNAs. *Nature*.2018;553(7686) : 10.1038/nature25027 82-85 10.1038/nature25027 29300014

[2] : microRNA-directed phasing during trans-acting siRNA biogenesis in plants. *Cell*.2005;121(2) : 10.1016/j.cell.2005.04.004 207-21 10.1016/j.cell.2005.04.004 15851028

# Response

**Published:** 2020-03-27

**Authors:** Christopher Rock

**Affiliations:** Texas Tech University, USA

Author responses to verbatim copied comments from Reviewer2 below, in conjunction with revised article submission.

“Analysis of Chen et al’s A. comosus MD-2 cultivar leaf small RNA (sRNA) libraries and stranded RNA-seq libraries from flowers and fruits of F153 and CB5 genotypes establish the existence of aco-miR828 (Extended data: Figure S1) and pri-MIR828 expressions, with the novel observation that pri-MIR828 is properly transcribed in leaves, flowers and fruits yet ~50% of mature miR828 species abundance (0.3 reads per million in leaves; Extended data: Table S1) are 21 nt, while 93% of the equally abundant 22 nt species appear to have undergone non-templated 3’ uridylation of the 21 nt species (Extended data: Table S1).”  --- This sentence does not clearly describe what “analysis” has taken place. I need to see the author describe what evidence supports the 3 conclusions listed here, both in writing and with a description of the analysis pipeline in the methods section (see below).

**RESPONSE: I thank the Reviewer for thorough and careful review and suggestions for improvement. Journal proscription on word limit (1,000) for the manuscript body (including Methods) was the reason for the (overly) brief description(s). Here is a pedantic description of the logical analyses employed to make the three referenced aco-MIR828 expression conclusions. Conclusion 1 “existence of aco-miR828 (Fig. S1)”: is from two independent ShortStack expression analyses detailed in Suppl. Table S1; i) mapping of sRNAs to the candidate aco-MIR828 locus/primary-MIR828 transcript corresponding to ShortStack Cluster_43525 in the new improved F153 reference genome, and ii) phasing of secondary siRNAs mapped as clusters to target MYB mRNA/cDNAs (older F153 annotated genome) triggered by inferred aco-miR828-programmed AGO slicing activity. Fig. S1 shows default ShortStack graphical aco-MIR828 hairpin secondary structure; the legend refers to Suppl. Table S1 for ShortStack tabular output details. Those tabular outputs on row 11 (primary-MIR828 Cluster_43525, length 333 nt) shows “Major RNA species”= UCUUGCUCAAAUGAGUAUUCCU, which is grounds for the claimed 3’ non-templated uridylated species (see elaboration below in Conclusion3 response). Tabular and graphical ShortStack outputs in Suppl. Table S1 and Fig. S1, respectively, are intended to document parameters required, according to community standards, for *MIRNA* annotation (Axtell MJ and Meyers BC [2018] “Revisiting criteria for plant microRNA annotation in the era of Big Data” *Plant Cell* 30: 272–284). Suppl. Table S1 rows 13 and 14 document ShortStack parameters of phased secondary siRNA clusters derived from two MYB target mRNAs/cDNAs: row13 “MajorRNA species” is D2(+) 21mer in phase (phasing score=20.2) with miR828 slice site activity independently substantiated by CleaveLand degradome analysis detailed in Fig. 1 (left panel). Furthermore, Fig. S2 further substantiates independent algorithmic evidence by PhaseTank software for the siRNA phasing on both MYB mRNA/cDNA targets triggered by miR828 activity. Suppl. Table S1 Row 14 “MajorRNA species”= CUAGGAGUGAAAGAUUACGCC was subsequently found (and annotated as such in a revised Suppl. Table S1) to map exactly 12 registers downstream from the 10 ^th^ nt position of D2(-) phasiRNA shown in Fig. S2 for mRNA/cDNA Aco020986. Thus, this additional subsequent analysis supports that Aco020986 D2(-) phasiRNA may analogously program AGO for secondary slicing of Aco020986 mRNA/cDNA and help explain the low ShortStack phasing score of 2.9 for Aco020986. Another plausible reason for the low phasing score of Aco020986 versus Aco017254 is the ~50% fewer reads (drawn from the multi-mapper functionality of ShortStack, parameter “RPM”) and higher reads complexity for Aco020986 per ShortStack output parameters. Notwithstanding, CleaveLand (Fig. 1 right panel) degradome and PhaseTank algorithmic analyses (Suppl. Fig. S2) independently show** **Aco020986 MYB mRNA/cDNA is a target of aco-miR828, substantiating not only aco-miR828 existence from expression data, but also its function.**

**Conclusion 2 “existence [and proper transcription] of pri- *MIR828* expressions [by analysis] of stranded RNA-seq libraries from flowers, and fruits of F153 and CB5 genotypes”: See Suppl. Table S1, rows 17-52 output parameters from MagicBLAST analysis. Row 22 is singular evidence of an RNA-seq read in genotype F153 leaf tissue (not formally claimed). Rows 23-36 are reads from F153 ovules and stamens. Rows 37- 52 are reads from genotype CB5 fruit.**

**Conclusion 3 “~50% of mature miR828 species abundance are 21 nt, while 93% of the equally abundant 22 nt species appear to have undergone non-templated 3’ uridylation of the 21 nt species”: See above re: row 11 of Suppl. Table S1 that establishes the MajorRNA species expressed in the sRNA libraries is the hypothesized non-templated 22 nt aco-miR828. Note that ShortStack output reports five reads of 20 nt size, 29 reads of 21 nt size, and 35 reads of 22mer size. Thus at face value assuming all species of these sizes correspond to mature miR828s (see below), then 51% would be the miR828 22mer species (other ShortStack-reported 23 and 24 nt sizes are few and discounted, yet documented in Suppl. Table S1). Rows 54- 136 of Suppl. Table S1 detail every sRNA read in the subject libraries sorted by position having at most one mismatch aligned by bowtie to the F153 primary-MIR828 locus corresponding to ShortStack full Cluster_43525, including 113 nt upstream and 97 nt downstream of the miR828 hairpin shown in Suppl. Fig. S1 as default ShortStack output. Each relevant read is annotated manually as 20mer mature miR828, 21mer mature miR828, 22mer perfect match, or 22mer non-perfect matches to mature miR828. There are four 20mer mature miR828, 26 21mer mature miR828, one perfect match mature miR828 22mer, one 22mer with a “C” mismatch at nt 22, and 28 22mers with “T” mismatch at nt 22 of miR828 that is the claimed non-templated 3’ uridylated species. Thus (26 +4)/(26+4+30) = 50% 20mer plus 21mer mature miR828 (my claim of “~50% 21 mer”), and 93% of 22mer species are 3’ uridylated (28/30). It takes >700 words here to pedantically articulate the grounds that prove the compound claims in that one sentence of the manuscript text.**

It is preferred to include a genome-browser shot showing the read coverage for the pri-miRNA regions listed in supp figure 1, as it can be difficult to identify from a table what the locus actually looks like for the reader. It also appears that many of the rna-seq reads originate from the ‘minus’ strand. This is surprising, as the hairpin is never double stranded. Could you explain this discrepancy?

**RESPONSE: I have modified Suppl. Fig. S1, keeping the default ShortStack dataset graphical output (instead of the suggested browser screenshot) but adding rows for species to visualize all relevant data described above for bowtie output in Suppl. Table S1, rows 57-136, where parameters of strand and importantly sRNA read location on the primary MIR828 transcript are detailed, along with manual annotations (including for nine anti-sense sRNA reads mapping only up- and downstream of the hairpin per se). Note also that formally speaking, the Reviewer’s reference to the ‘pri-miRNA regions’ at issue is most analogous to the “pre-MIRNA” after ‘stem-to-loop’ biogenesis as described in Adams, L. [2017] “Pri-miRNA processing: structure is key.” *Nat. Rev. Genet.* 18: 145) which is the default output of ShortStack software showing only the minimal foldback encompassing expression reads for mature miR828 and miR828-star. Suppl. Fig. S1 was verbatim output from ShortStack software that automatically trims the foldback stem distal to the miRNA/miRNA-star duplex. In this matter the full length cluster #43525 corresponding to the ‘primary-MIRNA’ transcript is described in Suppl. Fig. S1 image header as “Cluster_43525 Original Location: CABGUK010000004.1:12043665-12043997 Displayed Location: CABGUK010000004.1:12043762-12043883 Strand: -“. Therefore the graphical presentation in Suppl. Fig. S1 represents those pertinent sRNA species detailed in the Suppl. Table S1 list of reads corresponding to pri-MIRNA transcript positions 114 to 235. The bowtie-mapped reads described in Suppl. Table S1, rows 57- 136 sorted by primary-MIR828 position are now fully accounted for in the revised Suppl. Fig. S1 which retains the graphical default ShortStack output style. I added to the original Suppl. Fig. S1 four additional rows for the other species that are germane to the issue (excluding only four 23mer reads that have non-templated “CU” residues at the 3’ terminus of miR828, where the second “U” residue could be templated thus confounding description). Now both graphical (revised Suppl. Fig. S1) and tabular representations (Suppl. Table S1) quantify the principal species at issue: the 20 and 21 nt mature miR828 (~50%), the 22 nt mature miR828 with either a non-templated “T” as the last residue (lower case; ~50% of all reads), a non-templated “C”, or a perfect match 22 nt template mature miR828, and a single phased read (in the terminal loop) upstream of the predicted miR828-star species (shown all lowercase).**

**Regarding the RNA-seq antisense issue, note that row 20 of Suppl. Table S1 has a disclaimer description which the Reviewer may have overlooked: “Read 2 is the confirmed sense mRNA strand for CB5 datasets, based on an abundant transcript (bromelain RD21A, Aco011478.1) described by Chen et al. authors, used as control. The F153 ovule and stamen libraries are unstranded, based on the same control gene assessment.” It was deduced empirically from the RD21A reference gene characterization that the authors used un-stranded RNA-Seq kits early in the chronology of the project when many of the F153 ovule and stamen libraries were prepared and sequenced using different Illumina platforms. As evidence to support this interpretation, note there only exists a forward strand “read 1” for leaf, ovule, and stamen SRA datasets** **ERR3413090 and ERR3413005-8 (most are read lengths 1 x 100 bp, supporting their earlier chronology), despite being incorrectly annotated at NCBI SRA archive as “paired read” libraries. Specifically, rows 27, 28, and 36 are antisense strand reads challenged by the Reviewer and accounted for in the row 20 note as a computational artifact, thus counted as proper sense reads based on evidence from a control gene RD21A. For rows 38, 39, 46, 48, and 51, which are from CB5 fruit tissues (SRA datasets ERR3412993-3413002; read lengths 2 x 150 bp indicating newer Illumina chemistry and chronology) the libraries are verified empirically by expression evidence to be stranded paired read libraries. Note that in these antisense read cases, every read is from the antisense direction (read 1) of paired reads, as described in the row 20 disclaimer note.**

I like that the ShortStack output is included in supp. table 1. However, it is not clear to me what each locus refers to. What is the reference genome for each of these? It appears that cluster_1381 and cluster_1104 are using the Chen et al. sequencing data on the older reference genome. Why have these been shown and can you explain the lack of this locus in the older genome? I’m not sure what these loci are referring to compared to the first locus shown (cluster_4352).

**RESPONSE: as described above for “Conclusion1” evidence, row 11 aco-MIR828 ShortStack output is based on the new improved F153 reference genome made available as BioProject accession code PRJEB33121. ShortStack output for Rows 13 and 14 clusters were drawn from the cDNA fasta file of the original F153 reference genome available for download in phytozome12 as described in Data availability. Thus the two ShortStack analyses are different and cluster numbers are arbitrary. The reason why the older cDNA reference set from phytozome12 was used is because the new improved F153 reference genome does not have any cDNA annotation reference set. See https://ftp.ncbi.nlm.nih.gov/genomes/all/GCA/902/162/155/GCA_902162155.1_F153/.**

Your RPMs for this miRNA locus are extremely low (0.3 RPM), more than 10 fold lower than ShortStacks default locus identification threshold (5 RPM). This is a real lack and makes it challenging to identify this as a miRNA. Lacking a mir-star is also a problem, but might not be surprising for a low-expression locus. As this is a published miRNA you are identifying, I am inclined to agree that it is real. However, it is inappropriate to include an artificial sequence to make ShortStack call it as such, as is done in Supp. Figure 1. Please correct this and clearly identify that this locus lacks mir-star support, though is still likely a miRNA.

**RESPONSE: I have revised Suppl. Fig. S1 legend to state “This locus formally lacks miR828-star support.” Suppl. Fig. S1 legend clearly explained the miR828-star species was not sequenced from the dataset, but rather was seeded manually to drive ShortStack to formally call the MIRNA as “Yes” by default parameters, instead of “N15” in which case no graphical ShortStack output like Suppl. Fig. S1 is possible. It is argued that the thought experiment as described and presented is appropriate, if it can be justified. The Reviewer will recognize, as one skilled in the art of ShortStack, that without seeding a miR828-star sequence into the dataset, there would be no graphical ShortStack output at all, now revised (see above point) to meet the Reviewer’s specification to show the bona fide small RNA reads topology and abundances. The justification for the miR828-star “thought experiment” was to ensure annotation took into account default folding and trimming parameters built into the software where outputs are styled to objectively meet community standards for evidence-based MIRNA annotation. The reason for the exercise was not to obfuscate the truth that the star species was not found (which is clearly disclosed and the Reviewer concedes is not fatal because this is deeply conserved plant MIRNA), but rather to address a potential criticism that the “bulged” distal loop tripartite secondary structure (Suppl. Fig. S1) is an atypical feature of MIRNA foldbacks that could be an important determinant for Dicer recognition as substrate. This concern is grounded in the possibility a plant-specific “terminal loop-to-base” non-canonical processing sequence operates on aco-MIR828, like known for ath-miR156a biogenesis. Candidate aco-miR828 has a loop-side bulge analogous to the three nt loop-side bulge in ath-miR156a which appears as a “kink” in the foldback and could be argued as potentially critical for duplex biogenesis (Kim et al. [2016] “Structural determinants of miR156a precursor processing in temperature-responsive flowering in Arabidopsis.” *J. Exp. Bot.* 67: 4659). Being able to pass all algorithmic criteria as demonstrated by successful ShortStack annotation of candidate aco- *MIR828* (conceding the miR828-star aspect) discounts theoretical concerns about a “bulged” distal loop tripartite secondary structure possibly antagonizing Dicer duplex biogenesis at the locus.**

Consider looking into the package strucVis, an add-on for RNAfold which shows nucleotide-resolution read depth on the folded diagram. I think this would greatly clarify this figure.

**RESPONSE: I have revised Suppl. Fig. S1 to add the suggested strucVis image of small RNA read depth coverage across the aco-primary- *MIR828* locus encompassing the entire ShortStack reads Cluster_43525.**

“Analysis of independent bracteatus cultivar leaf sRNA libraries (NCBI SRA SRR5677552-7; 113.2 million reads) of unknown provenance relative to the subject CB5 genotype failed to identify any MIR828 reads.”  --- This is surprising! Did you also try analysis using the F153 reference, considering the provenance of this variety is unknown? I think this is important considering the considerable depth of these libraries, where we might expect around 30 reads coming from this locus. Despite the lack of a locus identified, it might be useful to look directly at the alignment through a browser, to see if any small RNAs are coming from this locus.

**RESPONSE: Note that rows 18 and 19 of Suppl. Table S1 state: “subject database F153 pri-MIR828 genome coordinates: CABGUK010000004.1:12043665-12043997rc; subject database CB5 pri-MIR828 genome coordinates: CABWKS010000025.1:21332774-21333104rc.” I have compared these two sequences by BLASTing the entire F153 aco-MIR828 locus and they are identical except for a single nucleotide polymorphism on the 3’ leg of the foldback, far downstream of the miR828-star at position 195 of the alignment (mature miR828 and miR828-star underlined). Thus the F153 and CB5 loci are identical for the purposes of running ShortStack and mapping small RNAs from bracteatus genotype sources.**

Please see here. 

**I have also subsequently run the strucVis software on the CB5 bam-indexed ShortStack output, as suggested to look directly at the alignment: there is no evidence of any reads mapping from the independent bracteatus cultivar leaf sRNA libraries to the CB5 MIR828, as claimed (data not shown). This is despite ShortStack calling 52 bona fide MIRNAs (47 21mers, five 22mers), compared with 29 MIRNAs (24 21mers, five 22mers) called with the F153 small RNA dataset.**

“...mature miR828 species abundance (0.3 reads per million in leaves; Extended data: Table S1)...” --- Please show the approximate read depth here also, as was shown with the Xiong et al libraries.

**RESPONSE: The answer is 264.7 million reads, available in Suppl. Table S1, row 5; that information has been added accordingly to the text.**

Figure 1 is labeled as a degradome, when it is actually a plot of 5’ ends of sRNA alignments, thought to be secondary siRNAs. While I agree that secondary siRNAs can be strong evidence of targeting. However, this is entirely distinct from a degradome, which are derived from isolated mRNA degradation products. Please remove references of this as a degradome and correct the language to clearly describe this. This also should contain a citation for one of the following papers: where this evidence was used for confirming targeting (Shahid et al. 2018 ^1^), or evidence of secondary sRNAs as a product of miRNA targeting (Allen et al. 2005 ^2^). References

1. Shahid S, Kim G, Johnson N, Wafula E, et al.: MicroRNAs from the parasitic plant Cuscuta campestris target host messenger RNAs. Nature. 2018; 553 (7686): 82-85 Publisher Full Text

2. Allen E, Xie Z, Gustafson AM, Carrington JC: microRNA-directed phasing during trans-acting siRNA biogenesis in plants.Cell. 2005; 121 (2): 207-21 PubMed Abstract | Publisher Full Text

**RESPONSE: I have revised the text as advised, calling inputs ‘pseudo-degradome’ amplified sense and antisense siRNAs derived from miRNA-sliced mRNAs, and citing those two priority references in addition to the 2019 self citation #7 where we coined the term pseudo-degradome in a study of vvi-miR828 activity.**

Figure 2 has several critical problems. In general, I think the author must provide more detail in the conclusions and evidence shown in each section of the figure. I have detailed some more specific points as follows:

It is deceiving to use bar plots to represent such small numbers of data points. Bars that represent an N of ideally 5 or less are much more informative shown as points representing the actual data. To show error bars (I assume... it is not indicated if these are standard error or deviation) greatly misrepresents the variance of these data. I would like to see these figures redone showing points rather than bars, or at least showing the raw data in a supplementary table (see below).

**RESPONSE: A factor perhaps not considered by the Reviewer when asking for “whisker plots,” i.e. drawn from separate data points: note that library size/depth determine normalized data points per library sample, summed within each library and reported as the mean of three biological replicate libraries. Thus only three such data points could possibly be shown for such a requested whisker plot, despite the underlying basis being many independent data points making up the normalized reads per library. Error bars were expressly described as standard error of three biological replicates in the legend of Fig. 2. The legend has been revised to direct readers to the raw data which was provided in Suppl. Table S1 rows 23-36 for MIR828 (panel A: 11 independent data points for ovules, 3 data points for stamens), and Suppl. Table S2, rows 406-470 for MYB mRNAs (panel A: 11 independent data points for Aco017254 ovules, 28 for stamens; 3 data points for Aco020986 ovules, 11 for stamens). It is rebutted that bars are thus not representing N < 5 data points in Fig. 2A as critiqued except for two: MIR828 stamens and Aco020986 ovules. It is conceded the error bars are not compelling statistically, but pointed out that no claims are made for significance while maintaining data integrity by reporting observed biological variance as found in the data. The verbatim claim made from Fig. 2A data states: “evidence supports higher pri-MIR828 expression in F153 ovules concordant with lower target MYB Aco017254.1 mRNA levels.”  This qualified claim is grounded from 11 independent ovule MIR828 and Aco017254 data points, respectively, averaged across three biological replicates which is derivative of the experimental design as found and interpreted conservatively.**

Figure 2B is vague in its description of the test, and replicates involved. The legend describes an N=1 for all stages. If this is true, how can a statistical comparison be made? Stage 1 shows error bars, does this represent N>1? Additionally, precisely which samples are being grouped and compared here for the test? This must be made clearer.

**RESPONSE: The Reviewer is appreciated for due diligence and critical insights. Here are the raw independent reads data summarized from Suppl. Table S2, rows 134-405, which the reader is directed to in the revised legend for completeness: Aco017254 stages 1, 2, 3, 4, 5, 7 and 8 have respectively 38, 37, 45,11, 5, 6, and 26 data points; Aco020986 has respectively 6, 7, 7, 0, 0, 1, and 3 unique independent data points (not cross- mapped to homologous Aco017254) for stages 1-8. The statistical comparison is made by taking independent abundances for Aco017254 and Aco020986 in stage 1 MD2 fruit as one condition matrix, and stages 2-8 as the second paired condition matrix across both genes to conduct a paired two-sided Student’s t-test for significance; the result is *p* = 0.02. The legend has been revised to clarify as requested. Stage 1 “error” bars in question are actually calculated 95% confidence intervals for hypothetical significance if there were a second biological replicate (extant experimental dataset is n=1) by using an unpaired Student’s t-test and introducing a second matrix datapoint for each gene of a magnitude sufficient to give *p* = 0.05. That magnitude, determined empirically, is the boundary marked by the low error bar as the 95% percentile confidence interval for statistical significance. The purpose of the analysis is to graphically show by an independent estimation, consistent with the paired t-test actual result, that stages 2-8 means are all well below the lower error bar confidence interval for significance calculated for stage 1.**

The interpretation of results in Figure 2C is unclear. There is little to no textual description of what the author concludes from Figure 2 in general and it is mainly confined to one sentence where all three sub-figures are mentioned. This needs to be greatly expanded upon for me to understand what they perceive as the outcome of these analyses. I recommend including *all* of the raw data shown in these charts in supplementary tables in addition to a more clear demonstration of the data-points mentioned above. This allows the reader to clearly identify what is being shown in each.

**RESPONSE: Again it is noted the issue of terse description of interpretations was driven by publisher proscription on word limits. Here are the raw independent reads data summarized from Suppl. Table S1 rows 37-52 for MIR828 and Suppl. Table S2, rows 471-883 for CB5 Aco017254, referenced in a revised Fig. 2 legend: 8 MIR828 independent data points each for 1 month- and 2 month-old CB5 fruits, and 171 data points for 1 month old-, and 222 data points for 2 month-old Aco017254 expression in CB5 fruits. Here is restated a revised but nonetheless succinct paraphrased claim from the text that intends to clarify how to compare evidences in Fig. 2C with Fig. 2B: “there may be differences in expression and/or regulation of pri-MIR828, and/or target MYB Aco017254.1 between F153 yellow-fleshed versus CB5 red-fleshed genotypes…. there is a trend of lower expression of CB5 pri-MIR828 concordant with sustained higher abundance of Aco017254.1 target *MYB* (Figure 2C stage 7 versus stage 1) than seen in MD-2 during the ripening stage of red-fleshed CB5 genotype (compare Figure 2C stage 7 showing high CB5 Aco017254.1 abundance to Figure 2B stage 7 showing very low MD-2 Aco017254.1 abundance).”**

A description of methods is lacking. Though the sequencing data is derived from prior publications, the analysis performed in this paper is new. You need to describe the logical steps taken for each analysis, including processing, tools used in a step, the actual commands used, and data that is output. This would go a long way to clarifying the actual analyses they performed. This needs to be shown for RNA-seq analyses, sRNA-seq analyses, and identification of pri-miRNAs.

**RESPONSE: This issue was raised by the journal’s managing Editor at the submission stage and addressed by the author. A statement is added to the end of the Software availability and methods section “The options parameters used for various algorithms are detailed in *Extended data*: Tables S1-  S3.”**

# Peer review: Reviewer response for version 1

**DOI:** 10.5256/f1000research.24007.r58563

**Published:** 2020-02-19

**Authors:** Brian D. Gregory

**Affiliations:** 1 Department of Biology, University of Pennsylvania School of Arts and Sciences, Philadelphia, USA

The article presents new findings that support the existence of MIR828 in the pineapple genome and that this microRNA may be involved in regulating *MYB* target RNAs for silencing to affect fruit coloration differences between various pineapple species. The study is well described and provides some intriguing new insights that other plant scientists are likely to be interested in reading. The conclusions are well supported and these preliminary findings definitely hint that the hypothesis suggested by Dr. Rock that this microRNA – target interaction may affect fruit color in pineapple is definitely plausible. I only have minor suggestions for changes to the article which I list below:

- The phrase “MYB expressions” should be changed to “ *MYB* abundance” throughout the manuscript.
- The phrase “phasiRNA accumulations” should be changed to “phasiRNA accumulation”.
- The phrase “phylogenomics approaches” should be changed to “phylogenomic approaches”.

Are arguments sufficiently supported by evidence from the published literature or by new data and results?

Yes

Is the conclusion balanced and justified on the basis of the presented arguments?

Yes

Is the rationale for commenting on the previous publication clearly described?

Yes

Are any opinions stated well-argued, clear and cogent?

Yes

Reviewer Expertise:

Plant RNA biology

I confirm that I have read this submission and believe that I have an appropriate level of expertise to confirm that it is of an acceptable scientific standard.

# Response

**Published:** 2020-03-27

**Authors:** Christopher Rock

**Affiliations:** Texas Tech University, USA

Suggested changes made in revised article
